# Spontaneous pneumomediastinum and subcutaneous emphysema in a non-intubated COVID-19 patient: a case report

**DOI:** 10.11604/pamj.2021.38.37.27543

**Published:** 2021-01-14

**Authors:** Tahir Ahmed Jatoi, Anosh Aslam Khan, Osama Mohiuddin, Muhammad Saad Choudhry, Farah Yasmin, Sumeen Jalees

**Affiliations:** 1 Department of Internal Medicine, Dow University of Health Sciences, Karachi, Pakistan,; 2 Department of Surgery, Dow University of Health Sciences, Karachi, Pakistan

**Keywords:** COVID-19, pneumomediastinum, subcutaneous emphysema, emergency department, case report

## Abstract

The development of spontaneous pneumomediastinum and subcutaneous emphysema are few of the rare clinical manifestations observed in coronavirus disease-19 (COVID-19) patients which are yet to be fully understood. Most cases of spontaneous pneumomediastinum arise due to factors causing high intra-alveolar pressure. Herein, we report a case of a COVID-19 positive elderly male, who presented with spontaneous pneumomediastinum and subcutaneous emphysema unrelated to high-pressure ventilatory measures, detected on chest computed tomography (CT). Despite acute medical care, the patient progressed towards a more serious clinical course. Male gender and diffuse alveolar damage caused by COVID-19 seems to be the most relevant association in this case. However, we have enlightened other possible pathological mechanisms and their association with severity index of COVID-19.

## Introduction

Since the pandemic of coronavirus disease (COVID-19), computed tomography (CT) scan has become a diagnostic modality of choice, playing a pivotal role in detecting early features of lung infection and disease evaluation. The typical radiographic features of COVID-19 pneumonia include multifocal ground-glass opacities with patchy consolidations, with the predilection of lower lobe or posterior lung region [1]. However, evidence of unusual features like spontaneous pneumomediastinum and subcutaneous emphysema have also been reported, which are defined as the presence of free air in the mediastinum and subcutaneous tissues, respectively. Both are mostly self-limiting conditions which usually presents with dyspnea, chest pain and mediastinal crunch, also known as Hamman crunch, on heart auscultation [2,3]. Spontaneous pneumomediastinum and subcutaneous emphysema are rare findings in the general population with an incidence rate of 3.0 and 1.2 per 100,000, respectively, however, their incidence among COVID-19 patients are still unknown. Pneumomediastinum mostly develops due to sudden rise in the intra-alveolar pressure such as prolonged cough, emesis, Valsalva maneuver and positive pressure ventilation causing the alveolar rupture and dissection of air along bronchovascular sheaths, paving its way into the mediastinum and reaching potential spaces like pleura, pericardium and soft tissues of chest and neck [4,5]. However, previous epidemics of the severe acute respiratory syndrome (SARS) and Middle East respiratory syndrome (MERS) have shown up to 12% incidence of pneumomediastinum independent of barotrauma, which leads to the question if there are any innate component of coronavirus species leading to lung pathology and air-leak disorders [6]. To the best of our knowledge, we present the first reported case of spontaneous pneumomediastinum and subcutaneous emphysema in COVID-19 pneumonia, independent of mechanical ventilation, from a developing country and discussed the possible mechanisms of these complications.

## Patient and observation

**Figure 1:**
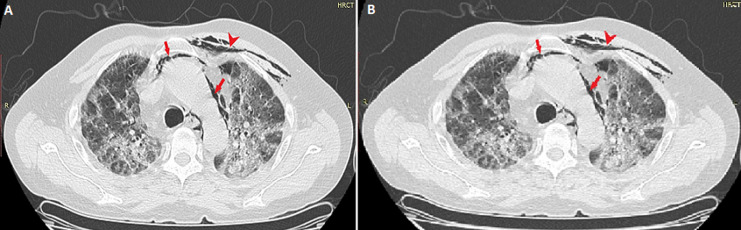
A, B) axial CT chest showing multifocal, bilateral ground glass opacities, predominantly in peripheral region and posterior lobes. Pneumomediastinum seen around aorta (arrows) and left paratracheal region. Subcutaneous emphysema is also visible (arrow head)

On 5^th^ October 2020, a 62-year-old male presented to the emergency department with 5-days history of low-grade fever, mild chest pain, dry cough, progressive fatigue and dyspnea. He was a non-smoker with a history of diabetes for 12 years. Upon arrival, he was conscious and alert with the respiratory rate of 36 breaths per minute, temperature 101° F, blood pressure 120/75 mmHg, pulse 102 beats per minute and arterial oxygen saturation (SpO_2_) was 93% at rest. On auscultation, bilateral coarse crepitation was audible throughout both the lung fields. A crunching sound, synchronous with the heartbeat, was auscultated at the cardiac apex. An area of subcutaneous emphysema was also felt on palpation of the left thoracic wall. Laboratory parameters revealed an elevated leukocyte count of 13 x 10^9^/L (normal 4 -10 x 10^9^/L). Both the erythrocyte sedimentation rate (ESR) and C-reactive protein (CRP) were elevated as follows: 75 mm/hr (normal: 0-20 mm/hr) and 390mg/L (normal: <5 mg/L), respectively. Serum ferritin and d-dimers levels were also raised at 1030 µg/L (normal: 13- 150 µg/L) and 1102 µg/L (normal: <500 µg/L), respectively. Lactate dehydrogenase (LDH) and procalcitonin levels were also raised at 528 U/L and 1.81 ng/mL, respectively. To confirm the high suspicion of COVID-19, coronavirus nucleic acid test (NAT) via real-time fluorescence polymerase chain reaction (RT-PCR) was ordered, which came out positive. Thereafter, the patient was admitted to the COVID-19 isolation ward on the day of the presentation. For the assessment of the severity of lung damage, a non-contrast CT of the chest was ordered, which revealed bilateral ground-glass opacities prominent posteriorly, with evidence of air lucencies around ascending and descending aorta. Subcutaneous emphysema was also observed in the left anterior thoracic wall. No pneumothorax or pleural effusion was seen (Figure 1 A, B).

The patient was given oxygen through a non-rebreather mask at 5L/min. At that time, his SpO_2_ was maintained at 94% at rest. He was started with the daily medication regimen of antiviral, antibacterial (lopinavir/ritonavir and piperacillin-sulbactam, respectively), thromboprophylaxis (enoxaparin 40 mg subcutaneous), hydroxychloroquine (400mg/ day) and intravenous (IV) methylprednisone 40 mg. For the next three days, his symptoms did not show any significant improvement except that his fever was subsided. During this time, he remained vitally stable except for the respiratory rate of 30 breaths per minute. The SpO_2_ kept fluctuating from 90% to 96%, which was strictly monitored and managed by maintaining oxygen through a non-rebreather mask at 8L/min to 5L/min. On 9^th^ October, his dyspnea suddenly exacerbated with SpO_2_ dropping to 84%. He remained hypoxic despite increased oxygen support on a non-rebreather mask at 15/L. By 10^th^ October, his SpO_2_ declined to 80%. The patient was switched to high flow nasal cannula at 50L/min and FiO_2_ 60%, for escalated oxygen support. Given his rapidly worsening oxygen status, the decision was made to transfer to the intensive care unit (ICU) on 12^th^ October. A second radiological chest imaging was planned to examine the status of spontaneous pneumomediastinum and subcutaneous emphysema, before going for intubation, however, the patient refused to undergo any imaging study. Nevertheless, the rapid response team was called and the patient was paralytically prone and intubated. During the ICU stay, his disease course was complicated by multi-organ failure requiring increased circulatory and ventilatory support. Despite maximum medical care, the patient, however, demised on 23^rd^ October.

## Discussion

Spontaneous pneumomediastinum is an uncommon clinical occurrence in the general population. It has been reported to be relatively prevalent among the young population with an incidence rate of 1/25,000 between the ages of 5 to 34 years. The majority of patients are male in more than 75% of the cases. Meanwhile, subcutaneous emphysema can also be detected in 70% of the patients of spontaneous pneumomediastinum. The clinical presentation of both spontaneous pneumomediastinum and subcutaneous emphysema can range from being asymptomatic to various symptoms including dyspnea, cough, dysphagia, and chest pain and voice changes [3]. Although by definition, spontaneous pneumomediastinum is considered idiopathic in origin, factors leading to intra-alveolar pressure such as prolonged coughing, chronic respiratory infection, asthma exacerbation, mechanical ventilation and emesis, are also included under the canopy of spontaneous pneumomediastinum. The rapidly developing high intra-alveolar pressure leads to rupture and release of air which dissects through pulmonary interstitium, along bronchovascular sheath towards pulmonary hila and reaching the mediastinal region. This pathophysiological mechanism is known as Macklin effect which was first described in 1944 [7]. For elderly patients like in our case, respiratory changes due to aging and pulmonary infections should be given primary attention. Increasing residual lung volume and decreased lung elastic recoil may disrupt pressure gradient leading to alveolar rupture. Furthermore, the incidence of pneumomediastinum in conjunction with pulmonary infections by pathogens like SARS, MERS and pneumocystis jiroveci has also been reported [6,8]. Henceforth, it is not surprising that COVID-19 pneumonia may also lead to the development of air-leak disorders like its predecessor, SARS. However, in various cases of viral pneumonia, spontaneous pneumomediastinum and subcutaneous emphysema have been noted as a complication of mechanical ventilation, which gives rises to the question of alternative pathological mechanisms [9]. Similar to SARS, COVID-19 virus has also been observed to cause diffuse alveolar damage (DAD), which leads to alveolar rupture and air leakage [9].

Moreover, recent literature has revealed that COVID-19 virus enters the target cell, including surfactant producing type II pneumocytes via angiotensin-converting enzyme-2 (ACE-2) receptors. These receptors are up-regulated in chronic hypertensive and diabetic patients, making this subset of the population more prone to the severe COVID-19 pneumonia. This postulation stands in concordance with our patient´s history, who was a non-smoker with no underlying medical conditions except a long-standing history of diabetes. Meanwhile, damage to type II pneumocytes hampers the production of the surfactant, impairing lung compliance which leads to hypoxia and promotes the risk of spontaneous pneumomediastinum (SPM) and subcutaneous emphysema (SE) development [10,11]. For diagnosis of spontaneous pneumomediastinum and subcutaneous emphysema, a plain anterior chest film can yield positive findings in 90% of cases, followed by chest CT scan for assessment of the extent of pneumomediastinum. CT scan is also sought as a primary diagnostic modality by physicians in cases of inconclusive chest X-ray. However, chest CT is considered the main tool for detection of COVID-19 and analysis of pulmonary pathology, with sensitivity up to 97%, hence it was also the first choice of diagnostic modality for our patient [2,12]. Typically spontaneous pneumomediastinum is considered as a benign entity, usually managed with rest, oxygen and analgesia [13]. Nevertheless, meticulous monitoring is mandatory to avoid possible complications like tension pneumothorax or cardiac tamponade [2,8]. However, in the light of SARS epidemic, the occurrence of spontaneous pneumomediastinum, independent of mechanical ventilation is related to increased risk of intubation and high mortality index, making it a potentially poor prognostic factor in cases of viral pneumonia [5]. Manna *et al*. studied eleven non-intubated patients of COVID-19 with spontaneous pneumomediastinum and subcutaneous emphysema. All these patients were non-smokers, among which five were diabetic and four patients expired during their hospital stay [14]. This is similar to the prognosis and data of our patient. Henceforth, it is vital to cautiously monitor COVID-19 patients in cases of spontaneous pneumomediastinum and subcutaneous emphysema and study risk factors and preventative measures for such complications.

## Conclusion

Spontaneous pneumomediastinum and subcutaneous emphysema are rare complications of COVID-19 pneumonia which have a possible association with disease severity. This necessitates further research about their risk factors, pathophysiology, preventative and management measures for better outcomes of the infected patients.
